# Phosphorylation of the actin-binding protein profilin2a at S137 modulates bidirectional structural plasticity at dendritic spines

**DOI:** 10.3389/fcell.2023.1107380

**Published:** 2023-02-15

**Authors:** Jonas Cornelius, Stefan Haak, Martin Rothkegel, Martin Korte, Kristin Michaelsen-Preusse

**Affiliations:** ^1^ Division of Cellular Neurobiology, Zoological Institute, TU Braunschweig, Braunschweig, Germany; ^2^ Helmholtz Centre for Infection Research, Research group Neuroinflammation and Neurodegeneration, Braunschweig, Germany

**Keywords:** synaptic plasticity, profilin, actin cytoskeleton, LTP, LTD

## Abstract

**Background:** Synaptic plasticity requires constant adaptation of functional and structural features at individual synaptic connections. Rapid re-modulation of the synaptic actin cytoskeleton provides the scaffold orchestrating both morphological and functional modifications. A major regulator of actin polymerization not only in neurons but also in various other cell types is the actin-binding protein profilin. While profilin is known to mediate the ADP to ATP exchange at actin monomers through its direct interaction with G-actin, it additionally is able to influence actin dynamics by binding to membrane-bound phospholipids as phosphatidylinositol (4,5)-bisphosphate (PIP2) as well as several other proteins containing poly-L-proline motifs including actin modulators like Ena/VASP, WAVE/WASP or formins. Notably, these interactions are proposed to be mediated by a fine-tuned regulation of post-translational phosphorylation of profilin. However, while phosphorylation sites of the ubiquitously expressed isoform profilin1 have been described and analyzed previously, there is still only little known about the phosphorylation of the profilin2a isoform predominantly expressed in neurons.

**Methods:** Here, utilizing a knock-down/knock-in approach, we replaced endogenously expressed profilin2a by (de)phospho-mutants of S137 known to alter actin-, PIP2 and PLP-binding properties of profilin2a and analyzed their effect on general actin dynamics as well as activity-dependent structural plasticity.

**Results and Discussion:** Our findings suggest that a precisely timed regulation of profilin2a phosphorylation at S137 is needed to mediate actin dynamics and structural plasticity bidirectionally during long-term potentiation and long-term depression, respectively.

## 1 Introduction

Neuronal plasticity and proper neuronal function depend on structural and functional changes at synapses which are based on rapid re-modulations of the synaptic actin cytoskeleton (Okamoto et al., 2004). Among the large number of actin regulators engaged in the subcellular organization of synaptic actin are profilins. Profilins catalyze the ADP-to-ATP exchange on G-actin (Goldschmidt-Clermont et al., 1992) and additionally interact with a plethora of other actin mediators as well as phospho-lipids to regulate actin dynamics in a multifaceted manner (Jockusch et al., 2007). In the mammalian CNS, two profilin isoforms are expressed, profilin1 (PFN1) and profilin2a (PFN2a) (Ackermann and Matus, 2003; Neuhoff et al., 2005), with PFN2a being the predominant isoform in neurons (Witke et al., 1998). Notably, both isoforms share redundancies in their subcellular localization as both are located at pre- and postsynaptic sites (Neuhoff et al., 2005) as well as in the nucleus (Birbach et al., 2006). In addition, an activity-dependent translocation into dendritic spines could be shown for both, PFN1 and PFN2a (Ackermann and Matus, 2003). Non-etheless, both isoforms were shown to be differentially engaged in the regulation of synaptic actin as PFN1 was found to be important for dendritic spine formation (Sungur et al., 2022) while PFN2a was shown to be involved in spine stabilization, synaptic function as well as activity-dependent structural plasticity (Michaelsen et al., 2010a; Michaelsen-Preusse et al., 2016). In line with this, although both isoforms share certain functional redundancies, the loss of one isoform cannot be fully compensated by the other (Michaelsen-Preusse et al., 2016).

In the cellular context, PFN function is mediated through its interaction with other proteins as, apart from its actin-binding pocket, PFNs possess binding sites for proteins containing polyproline-stretch (PLP-) motifes as well as membrane-bound phospholipids (Jockusch et al., 2007). Consequently, PFNs interact with a variety of other proteins involved in the regulation of structural and functional properties at synapses, including WAVE/WASP, Ena/Vasp, formins, ARP2, ARP3, Gephyrin or phosphatidylinositol-4,5-bisphosphate (PIP_2_) (Lassing and Lindberg, 1985; Machesky et al., 1994; Reinhard et al., 1995; Chang et al., 1996; Mammoto et al., 1998; Miki et al., 1998). Notably, PFNs can bind in parallel to actin as well as to proteins containing poly-(L)-proline stretches (e.g., formins) which, given the fact that poly-(L)-proline stretches are repetitive (Lambrechts et al., 1997), offers the possibility that PFN-actin complexes can be delivered and released with a high temporal and spatial resolution to sites of need, e.g., during phases of synaptic plasticity. On the contrary, the interaction between PFN and the membrane-bound PIP_2_ has been shown to dramatically reduce the affinity to PLP-proteins (Walter et al., 2020) indicating that PFN-PIP_2_-binding might serve as a regulatory mechanism to prevent PFN from interacting with actin. Hence, PFN availability as well as activity is fine-tuned through its interaction with other proteins.

Although the binding-partners of neuronal PFNs are in general rather well described, details about how these interactions are modulated in the cellular context are still missing. In this respect, post-translational modifications of PFN and especially PFN-phosphorylation seem to be of crucial importance. For PFN2, several phosphorylation-sites have been described that were shown to influence the affinity to other binding partners: 1) PFN2a phosphorylation at S71, S76 or S129 was shown to decrease actin-binding, 2) phosphorylation at Y78 was shown to increase PIP2 binding, 3) phosphorylation at Y29 or Y133 was found to decrease PLP-protein binding and 4) phosphorylation of S137, which is in close proximity to the PLP, PIP_2_ as well as actin-binding pocket and conserved among PFN isoforms, was shown to diminish binding to all binding-partners (Walter et al., 2020). Hence, targeted PFN2a (de)phosphorylation is likely to represent an effective mechanism mediating intracellular PFN2a function with a high degree of temporal as well as spatial resolution which might be especially important for processes of synaptic plasticity.

Therefore, to better understand the regulation of PFN2a in neurons, especially in the context of synaptic plasticity processes, we utilized a knock-down/knock-in approach to replace endogenously expressed PFN2a with phospho-mimetic or phospho-deficient PFN2a S137 mutants to elucidate the mode of action on basal actin dynamics as well as activity-dependent structural plasticity. Interestingly, our results suggest that a precisely timed phosphorylation- or dephosphorylation of S137 is needed to regulate spine plasticity and the underlying actin dynamics bidirectionally during NMDAR-dependent long-term potentiation (LTP) as well as NMDAR-dependent long-term depression (LTD).

## 2 Materials and methods

Generation of PFN2a gene replacement vectors. As already described previously (Michaelsen et al., 2010a; Schweinhuber et al., 2015), RNAi constructs were based on pRNATU6.3/Hygro (Genscript). For PFN2a-specific knockdown ds oligodeoxynucleotide, GAT​CCG​GAT​AAC​CTG​ATG​TGC​GAT GGC​GAA​CCA​TCG​CAC​ATC​AGG​TTA​TCC​TTT​T was inserted into the vector and the reporter EGFP was replaced by mApple (Kind gift of Michael W. Davidson, National High Magnetic Field Laboratory, USA). For gene replacements, synthetic cDNAs encoding RNAi-PFN2a-mod, RNAi-PFN2a-S137A or RNAi-PFN2a-S137D were ligated into the PFN2a-specific shRNA vector. The expression of mApple-PFN fusion proteins was driven by a truncated CMV promoter (Boshart et al., 1985).

Primary embryonic hippocampal cultures from C57Bl/6 wildtype mice were prepared at embryonic day 18 as described previously (Michaelsen et al., 2010a). 70.000 cells were plated on 13 mm poly-L-lysine coated coverslips and were maintained at 37°C, 5% CO_2_ (vol/vol) and 99% humidity in Neurobasal medium (Gibco) which was additionally supplemented with 10% N2 (vol/vol), 2% B27 (vol/vol) and 0.5 mM Glutamax (Gibco).

Transfection of cultured hippocampal neurons. Primary embryonic hippocampal cultures were transfected after 14 days *in vitro* using Lipofectamine 2000 (ThermoFischer Scientific) according to the manufacturer’s instructions. We used 2 µl of Lipofectamine and 1 µg plasmid-DNA as a combination of two of the following plasmids per well (24-well plate, 0.5 µg + 0.5 µg): EGFP-tagged ß-actin for fluorescence recovery after photobleaching experiments or farnesylated-EGFP to visualize individual neurons either combined with mApple (for controls) or combined with one of the PFN2a gene replacement vectors. Cultures were then used for experiments once PFN2a knockdown was complete—9 days after transfection (Michaelsen-Preusse et al., 2016).

cLTP induction in primary embryonic hippocampal cultures. All cells were incubated at room temperature for 20′ in 1x Hanks Balanced Salt Solution (HBSS, Invitrogen; 37°C pre-heated) and afterwards stimulated for 10′ using Mg^2+^-free HBSS containing 200 µM glycine and 3 µM strychnine. Immediately afterwards, the Mg^2+^-free HBSS was replaced with normal HBSS and cells were incubated for up to additional 50’.

cLTD induction in primary embryonic hippocampal cultures. All cells were incubated at 37°C, 5% CO_2_ (vol/vol) and 99% humidity in Neurobasal medium (Gibco) supplemented with 10% N2 (vol/vol), 2% B27 (vol/vol) and 0.5 mM Glutamax (Gibco) containing 200 µM NMDA for 10’ (Michaelsen et al., 2010b). Afterwards, all cells were incubated in similar environmental conditions for additional 50’ in the same medium without NMDA.

2D gel electrophoresis and immunoblotting. PFN2a was detected in hippocampal cell lysates. For 2D gel electrophoresis, the ZOOM IPGRunner System (Invitrogen) was used according to manufacturer’s instructions. Briefly, cells were lysed in HC buffer (8 M urea, 2% CHAPS, 0.003 M TRIS, 0.0625M DTT) with protease and phosphatase inhibitors as well as ampholytes and 15 µg total protein sample were used for isoelectric focusing. Afterwards, the strips were placed onto SDS-PAA gels and proteins were separated according to their molecular weight through SDS-PAGE before being blotted onto nitrocellulose membranes. PFN2a specific signals were detected *via* polyclonal rabbit anti-PFN2a primary antibodies (anti-PFN2a as361, 1:1000 (Murk et al., 2009)), anti-rabbit IgG-horse radish peroxidase coupled secondary antibody treatment (1:20.000 Sigma Aldrich A8275), subsequent HRP-substrate dependent signal amplification (Luminata Crescendo Western HRP Substrate) and X-ray film development. For analysis, intensity values of all PFN2a-specific spots were totaled and intensity values of individual dots were put into relation to total signal intensity.

Fluorescence recovery after photobleaching (FRAP). FRAP experiments were performed using primary embryonic hippocampal cultures (after 23 days in culture), in which EGFP-tagged ß-actin fluorescence was bleached in individual dendritic spines using the 405 nm laser line at a power of 2.3–3 mW (approx. 30%) for 150 ms. Simultaneously, EGFP emission at 488 nm was recorded using a SIM scanner (Olympus) and a time-lapse series was recorded for app. 180 s following photobleaching with a time interval of 3 s (65 images in total: five images pre-bleach, 60 post-bleach). All images were analyzed using ImageJ software. In brief, mean fluorescence intensity values for each time point were normalized to average intensities derived from 5 pre-bleaching images for each spine individually, and plotted against time. Subsequently, utilizing the GraphPad Prism software, non-linear curve fitting of net recovery curves after photobleaching were calculated using the following equation: Y = Y0 + (Plateau − Y0) × (1 − exp(−K × x)), where Y0 equals Y value at time point zero after bleaching. The plateau corresponds to the Y value at infinite times, and expressed as the fraction of fluorescence before bleaching. This value was used for determining dynamic and stable actin pools (the stable pool is the fraction of fluorescence that does not recover within the imaging period of 3 min calculated as 1 − (dynamic F-actin)), K is the rate constant, and τ is the time constant, expressed in seconds; it is computed as the reciprocal of K. From this equation, the actin turnover rate was calculated as the time point at which 50% of pre-bleaching fluorescence levels were reached.

Imaging and image analysis. Imaging was done with a confocal laser scanning microscope (Olympus Fluoview1000) equipped with 20x (0.5 NA), 40x (1.3 NA) and 60x (1.0 NA) objectives or a Leica TCS SP8 STED microscope equipped with 20x (0.75 NA), 93x (1.3 NA) and 100x (1.4 NA) objectives and resulting images were analyzed using ImageJ. For the analysis of dendritic spine properties, we quantified average dendritic spine head diameters as well as total number of dendritic spines (spine density: spines per µm dendrite) on given dendritic segments.

Generation of a PFN2a surface model. To generate a PFN2a surface model, the CCP4 Molecular Graphics Program was utilized (v. 2.10.11) (McNicholas et al., 2011). Based on an available X-ray structure of mouse PFN2a complexed with the PLP-domain of VASP (RCSB Protein Data Bank: 2V8C) and based on previous studies describing the respective binding sites of PFNs (Schutt et al., 1993; Krishnan and Moens, 2009), the PIP_2_ binding site (R74, R88, K90, K125, R136), the actin binding site (F59, V60, N61, K69, S71, V72, I73, R74, E82, R88, K90, T97, N99, V118, H119, G121, M122, N124, K125, Y128, E129) as well individual phosphorylable amino acids (Y29, Y78, Y133, Ser137) were highlighted and color-coded in CCP4MG for better visibility.

Data presentation and statistical analysis. If not specifically stated otherwise, data are depicted as means ± SEMs and an α level of *p* < 0.05 was used as criterion to reject the null hypothesis. Comparisons between two different groups were done using unpaired Student’s T-tests while comparisons of two or more groups were analyzed by One-Way or Two-Way ANOVA. A complete list of statistical tests and values can be found in Supplementary Table S1.

## 3 Results

### 3.1 Replacement of endogenous PFN2a with phospho-mutants alters hippocampal dendritic spine properties

**FIGURE 1:**
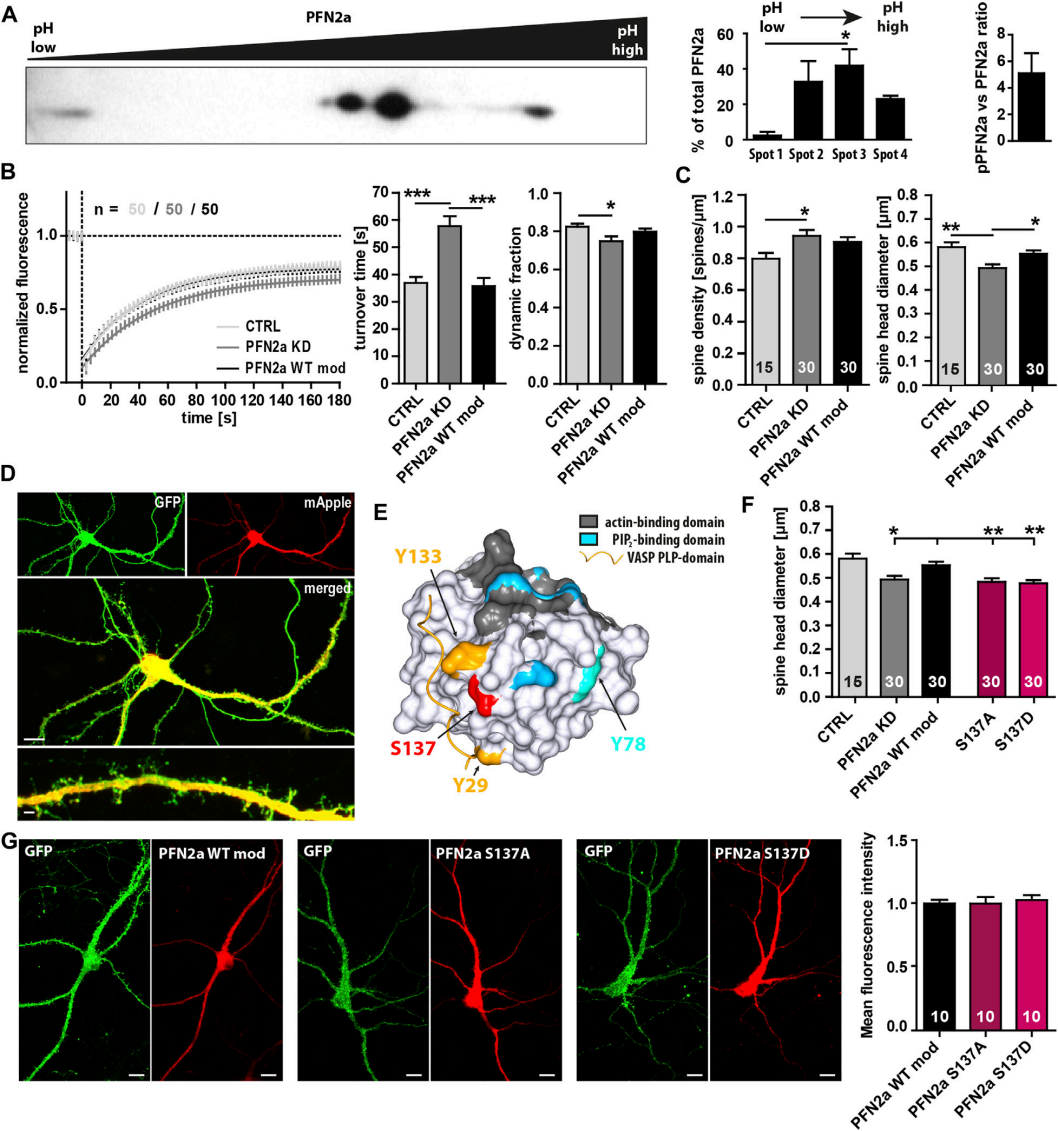
PFN2a modulates endogenous spine actin dynamics and dendritic spine morphology. **(A)** Separation of PFN2a by isoelectric point and molecular weight *via* 2D gel electrophoresis with subsequent Western blot analysis to depict the basal phosphorylation states of PFN2a. For voluminetric analysis of spot intensity N = 3 gels. **(B)** Fluorescence Recovery After Photobleaching (FRAP) experiments of EGFP-ß-actin depicting basal actin dynamics in hippocampal neurons after 23 days in culture. Depicted are the recovery curves over a time course of 180s after the bleaching pulse of hippocampal WT spines as well spines from neurons where PFN2a was knocked-down and replaced with either mApple (PFN2a KD) or wildtype PFN2a (PFN2a WT mod). In addition, the quantification of the turnover time as well as the fraction of dynamic actin is depicted (resembling the amount of fluorescence which recovered in the time period of 180s after the photobleach). *n* = 50 dendritic spines each. N = 3 experiments. **(C)** Analysis of dendritic spine density and dendritic spine head diameter in hippocampal WT control, PFN2a knockdown as well as PFN2a WT mod re-expressing neurons. *n* = 15, 30, 30 dendrites respectively (1 dendrite was analyzed per neuron). N = 3 experiments. **(D)** Example images depicting a hippocampal neuron where PFN2a was knocked down as indicated by expression of mApple. Scale bars - 10 µm (middle), 2 µm (bottom). **(E)** PFN2a surface model indicating the location of the known phosphorylable amino acids Y29 (yellow), Y78 (light blue), Y133 (yellow) and S137 (red) that were targeted for the generation of PFN2a phospho- and dephospho-mimetic mutants. To highlight their close proximity to known binding domains, the PFN2a actin-binding domain (grey), the PIP2-binding domain (blue) as well as the PLP-binding domain (indicated *via* a VASP PLP-domain, yellow) were marked as well. **(F)** Analysis of the average dendritic spine head diameter of hippocampal neurons where endogenous PFN2a was replaced with one of the different PFN mutants. *n* = 15 (Ctrl), 30 (KD), 30 (WT mod), 30 (S137A), 30 (S137D) (1 dendrite was analyzed per neuron). N = 3 experiments. **(G)** Analysis of mApple mean fluorescence intensity in the cell body of transfected hippocampal neurons where endogenous PFN2a had been replaced by PFN2a WT mod, S137A or S137D (9 days post-transfection). Example images depict individual neurons co-transfected with EGFP and the different mApple-tagged PFN2a variants. Scale bars - 10 µm.

Multiple phosphorylation sites of PFN2a have been described previously (Walter et al., 2020), however, the general phosphorylation patterns (Nolle et al., 2011), the molecular mechanisms mediating PFN2a (de)phosphorylation, and especially the potential impact of PFN2a phosphorylation on dendritic spine actin dynamics remain mostly elusive. Thus, at first, we performed 2D gel electrophoresis and separated different post-translationally modified PFN2a variants from hippocampal lysates dependent on their isoelectric point and molecular weight. Interestingly, under basal conditions, we found three different phosphorylation patterns (Figure 1A Spots 1–3, left to right) and ∼20% of PFN2a to be completely dephosphorylated (Figure 1A Spot 4) indicating that, in the hippocampus, PFN2a is predominantly present in a phosphorylated state with a ratio of approximately 5:1 between phosphorylated (pPFN2a) and dephosphorylated PFN2a. In addition, the main proportion of PFN2a was found to be present in two different phosphorylation patterns (Figure 1A Spot 2,3) while hyperphosphorylated PFN2a made up only a very small proportion (2.35% ± 2.13%, Figure 1A Spot 1). These observations suggest that PFN2a is regulated by phosphorylation *in vivo*, potentially at multiple phosphorylation sites simultaneously, indicating that distinct phosphorylation patterns of PFN2a might also serve individual roles in the cellular context by mediating PFN2a function. Thus, to shed light on the importance of PFN2a phosphorylation and individual PFN2a phosphorylation sites for dendritic spine morphology and function, we generated different mApple-tagged PFN2a phospho-mimetic and phospho-deficient mutants to use them in a knock-down/knock-in approach to replace endogenously expressed PFN2a in cultured hippocampal neurons. However, at first, as a control, we knocked down endogenous PFN2a and re-introduced unaltered wildtype PFN2a (PFN2a WT mod) to confirm that the knock-down/knock-in approach in itself had no effect on dendritic spine morphology and function in general. We analyzed basal synaptic actin dynamics *via* Fluorescence Recovery After Photobleaching experiments in EGFP-tagged ß-actin expressing hippocampal neurons which were either expressing mApple (Ctrl) or where endogenous PFN2a was knocked down and which were either expressing mApple only (pure PFN2a knock-down Ctrl ‘PFN2a KD’, Figure 1D) or an mApple-tagged, knock-down resistant PFN2a (PFN2a WT mod, Figure 1B). Notably, in comparison to mApple-expressing hippocampal control neurons, the knock-down of PFN2a altered basal actin dynamics prominently as it significantly increased the turnover time (the time needed for a 50% recovery of the initial fluorescence intensity) and significantly decreased the proportion of dynamic actin (which recovers within the imaging time window, Figure 1B). In addition, the knockdown of PFN2a led to a significant increase in dendritic spine number (Figure 1C) as well as a significant decrease of the average dendritic spine head diameter (Figure 1C). Importantly, however, all these phenotypes were rescued completely by re-introducing wildtype PFN2a (PFN2a WT mod, Figures 1B, C) indicating that the knock-down/knock-in in itself does not alter basal actin dynamics or dendritic spine characteristics. Thus, as a next step, based on previously identified PFN2a phosphorylation sites (Walter et al., 2020) (Figure 1E), we generated mApple-tagged PFN2a mutants mimicking a phosphorylation (PFN2a S137D) or a dephosphorylation at S137 (PFN2a S137A), a site close to all binding-pockets of PFN2a and known to influence binding kinetics to all binding partners. Interestingly, knock-down/knock-in using these mutants led to a significant decrease in dendritic spine head diameter, similar to what was observed following PFN2a knock-down (Figure 1F). Furthermore, to confirm that all PFN2a knock-in variants are expressed at similar rates, we analyzed the mean fluorescence intensity in the cell body of transfected hippocampal neurons 9 days after transfection (Figure 1G). Notably, fluorescence intensities were nearly identical for PFN2a WT mod, S137A as well as S137D.

### 3.2 Basal synaptic actin dynamics are influenced by PFN2a phosphorylation

**FIGURE 2:**
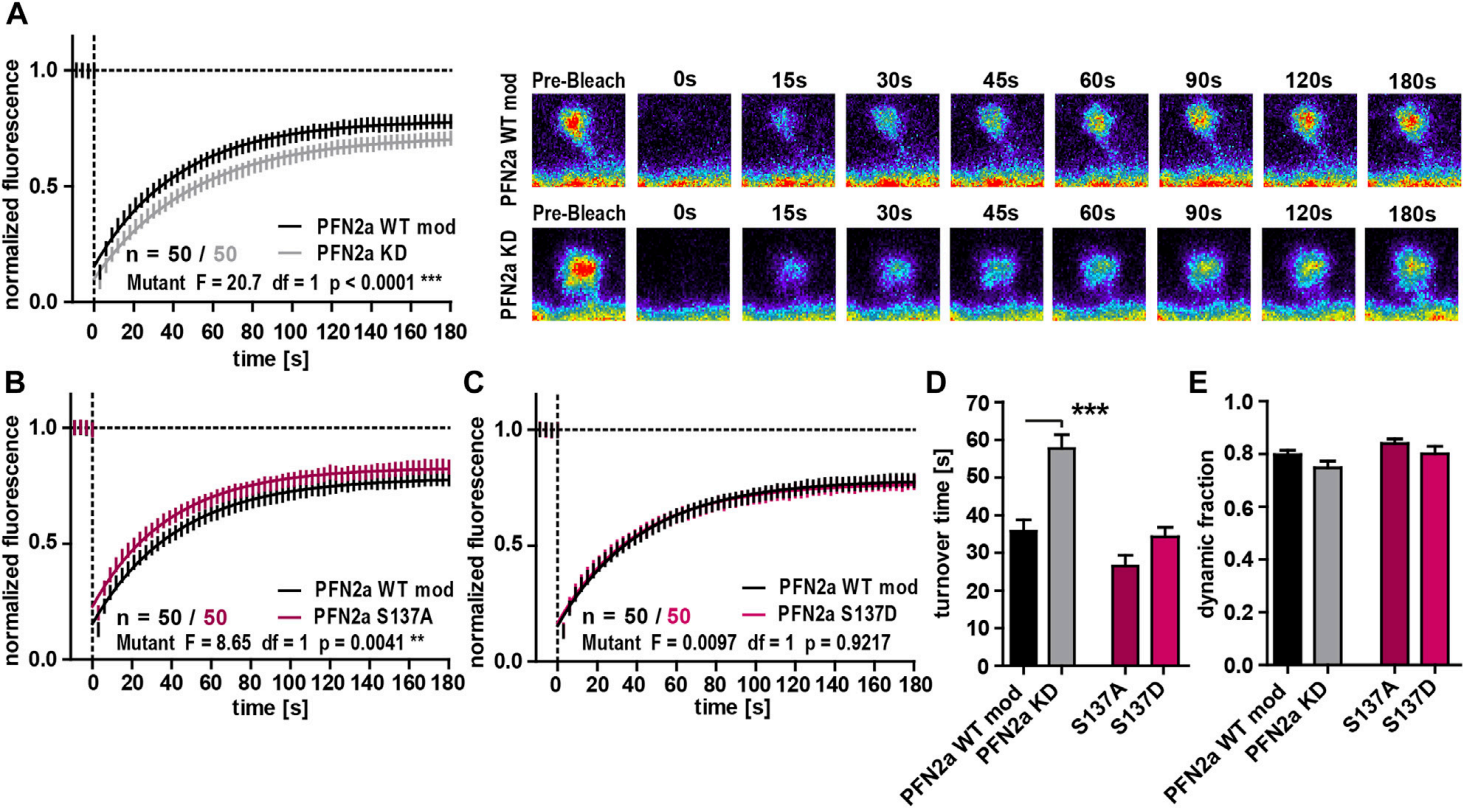
PFN2a regulates basal spine actin dynamics. **(A)** Fluorescence Recovery After Photobleaching (FRAP) experiments of EGFP-ß-actin analyzing basal actin dynamics in hippocampal neurons after 23 days in culture. Depicted are the recovery curves over a time course of 180s after the bleaching pulse of dendritic spines from hippocampal neurons where endogenous PFN2a was knocked down and replaced with WT PFN2a (PFN2a WT mod) or mApple (PFN2a KD). *n* = 50 spines each. N = 3 experiments. Representative images show fluorescence levels derived from EGFP-ß-actin of an individual dendritic spine before bleaching as well as during recovery of fluorescence over a time course of 180s after the bleaching pulse. Note that the data depicted in **(A)** is based on the same data already shown in Figure 1B. **(B, C)** FRAP experiments comparing basal actin dynamics in dendritic spines from PFN2a WT mod and **(B)** PFN2a S137A or **(C)** PFN2a S137D. N = 3 experiments. **(D)** Quantification of the turnover time. **(E)** Quantification of the amount of fluorescence which recovered in the time period of 180s after the photobleach—the fraction of dynamic actin.

As our previous experiments suggested that PFN2a phosphorylation has an impact on dendritic spine morphology, we were interested in a next step how (de)phosphorylation might influence basal spine actin dynamics as the underlying driving force for morphological changes (Matus, 2000; Honkura et al., 2008; Hotulainen and Hoogenraad, 2010). The knock-down of PFN2a led to a significant increase in the F-actin turnover time compared to PFN2a WT mod control cells (Figures 2A, F). In contrast to our results obtained for spine head diameter, actin dynamics were influenced differently by the PFN2a mutants. The F-actin recovery curve for cells expressing the phospho-deficient mutant PFN2a S137A showed a significant shift towards increased actin dynamics (Two-Way ANOVA F = 8.65, df = 1, *p* = 0.0041, Figure 2B), albeit no significant changes in F-actin turnover time or the dynamic actin fraction were found (Figures 2F, G). Actin dynamics were, however, completely unaltered in cells expressing phospho-mimetic PFN2a S137D (Figures 2C, F, G). The proportion of dynamic actin, however, was unaltered in both of the conditions that were tested (Figure 2G).

### 3.3 Phosphorylation at S137 is needed for early activity-dependent changes in dendritic spine actin dynamics following LTP induction

**FIGURE 3:**
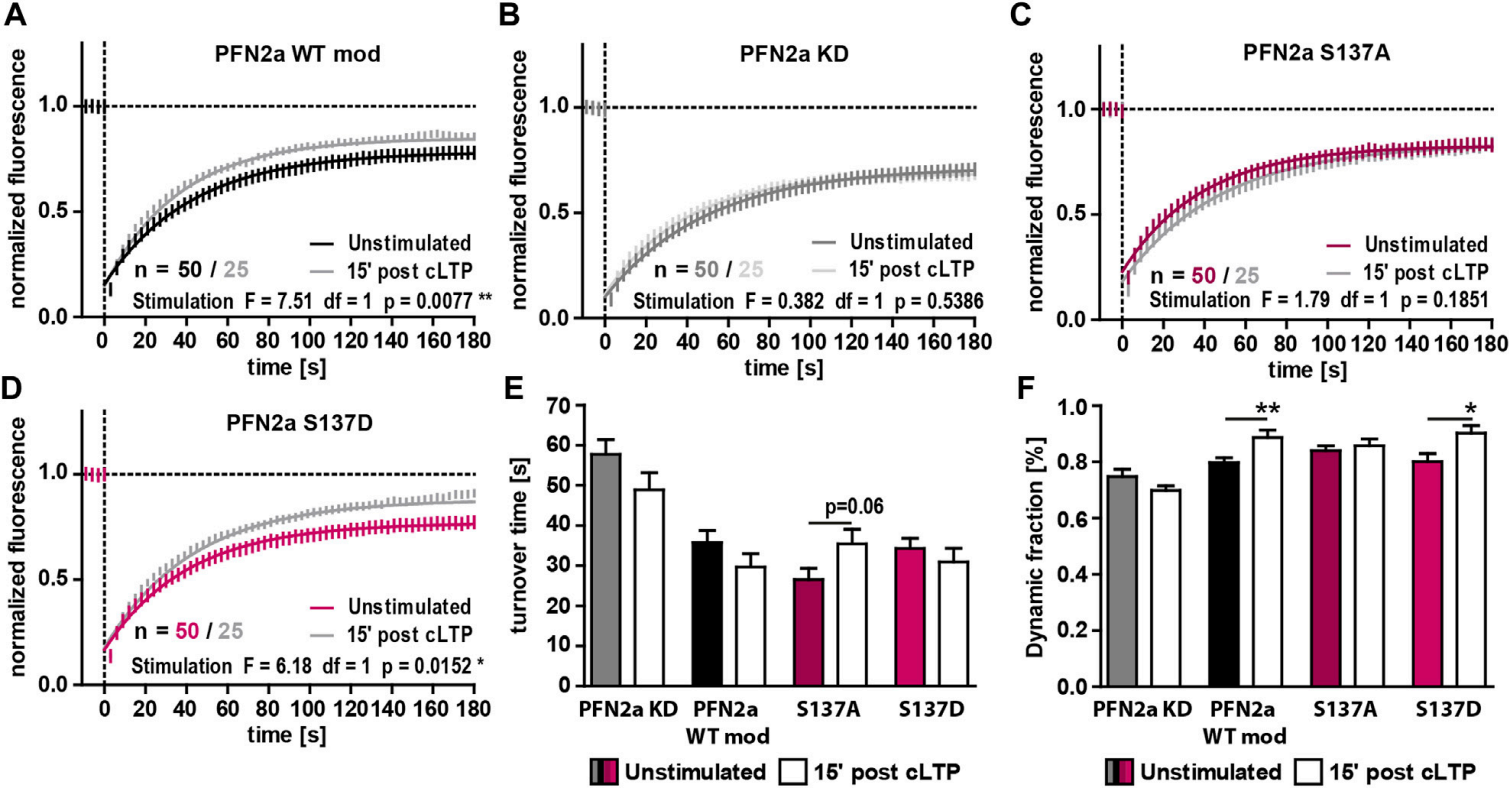
PFN2a phosphorylation at S137 mediates activity-dependent spine actin dynamics. **(A–D)** Fluorescence Recovery After Photobleaching (FRAP) experiments of EGFP-ß-actin analyzing actin dynamics 15′ post NMDAR-dependent cLTP induction in hippocampal neurons after 23 days in culture. Depicted are the recovery curves over a time course of 180s after the bleaching pulse of dendritic spines from hippocampal neurons where endogenous PFN2a was knocked down and replaced with **(A)** WT PFN2a (PFN2a WT mod), **(B)** mApple (PFN2a KD), **(C)** PFN2a S137A or **(D)** PFN2a S137D. *n* = 50 (Unstimulated), 25 (post cLTP) dendrites each. N = 3 experiments. **(E)** Quantification of the turnover time. **(F)** Quantification of the amount of fluorescence which recovered in the time period of 180s after the photobleach - the dynamic fraction of actin.

PFN2a was shown to be important for activity-dependent spine head growth following the induction of long-term potentiation (LTP) as well as for an activity-dependent modulation of spine actin dynamics (Michaelsen-Preusse et al., 2016). We therefore asked whether (de)phosphorylation at S137 might be one of the underlying mechanisms modulating dynamic actin during plasticity. We focused especially on the early phase following LTP induction (first 15’) as it is generally characterized by a very high degree of active F-actin remodeling (Okamoto et al., 2004; Chen et al., 2015). In line with this, the induction of cLTP in control neurons (PFN2a WT mod) led to a significant increase of dynamic F-actin (Figures 3A, E, F). These modulations, however, could not be seen in PFN2a deficient cells (PFN2a KD) highlighting the importance of PFN2a for activity-dependent spine actin dynamics (Figures 3B, E, F). Interestingly, substitution of endogenous PFN2a with phospho-deficient PFN2a S137A prevented activity-dependent changes of F-actin dynamics as well, as no alteration in the proportion of dynamic actin and even a tendency to rather increased F-actin turnover time could be observed (Figures 3C, E, F). Contrarily, in comparison to PFN2a WT mod expressing control neurons, F-actin dynamics following cLTP induction were unaltered in neurons where the phospho-mimetic mutant PFN2a S137D was introduced as also here, a significant increase in the proportion of dynamic actin was observed (Figures 3D–F).

### 3.4 Regulation of PFN2a S137 is crucial for structural plasticity following LTP as well as LTD induction

**FIGURE 4:**
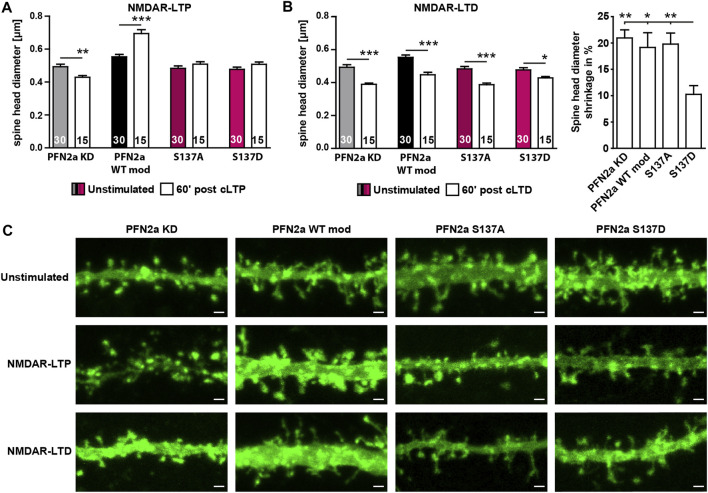
Regulation of PFN2a phosphorylation at S137 is essential for structural plasticity following NMDAR-dependent cLTP as well as cLTD induction. **(A)** Average dendritic spine head diameter 60′ post NMDAR-dependent cLTP induction of spines from hippocampal neurons where endogenous PFN2a had been replaced either by mApple (KD), WT PFN2a (WT mod), PFN2a S137A or PFN2a S137D. *n* = 30 (Unstimulated), 15 (cLTP) dendrites each (1 dendrite was analyzed per neuron). N = 3 experiments. **(B)** Average dendritic spine head diameter 60′ post NMDAR-dependent cLTD induction of spines from hippocampal neurons where endogenous PFN2a had been replaced either by mApple (KD), WT PFN2a (WT mod), PFN2a S137A or PFN2a S137D. *n* = 30 (Unstimulated), 15 (cLTD) dendrites each (1 dendrite was analyzed per neuron). N = 3 experiments. Note that, although spine shrinkage could be observed under all experimental conditions, when comparing the percentage of spine head shrinkage following cLTD induction, spine shrinkage was significantly reduced by ∼50% in neurons expressing the PFN2a S137D phospho-mimetic. **(C)** Example images depicting individual dendritic stretches from hippocampal neurons where endogenous PFN2a had been replaced either by mApple (KD), WT PFN2a (WT mod), PFN2a S137A or PFN2a S137D either unstimulated or 60′ post NMDAR-LTP or NMDAR-LTD induction. Scale bars - 1 µm.

Since our FRAP experiments indicated that phosphorylation of PFN2a at S137 indeed seems to be crucial for activity-dependent modulations of synaptic actin in the early phases of LTP induction, we tested next whether PFN2a S137 phosphorylation is also mediating spine structural plasticity following LTP as well. Therefore, we chemically induced NMDAR-dependent LTP in primary dissociated hippocampal cultures as before and analyzed the average dendritic spine head growth 60′ post LTP induction in neurons where endogenous PFN2a was substituted with wildtype PFN2a (PFN2a WT mod), mApple (PFN2a KD ‘knock-down control’), PFN2a S137A or PFN2a S137D. In line with already published data from our group (Michaelsen-Preusse et al., 2016), the induction of cLTP led to a significant increase in the average spine head diameter in PFN2a WT mod control neurons while the knock-down of PFN2a abolished spine growth and even led to significant spine shrinkage (Figures 4A, C). Interestingly, spine head growth was also prevented in either PFN2a S137A or PFN2a S137D expressing neurons.

As these results suggested that in order to mediate dendritic spine growth, PFN2a needs to be accessible for phospho- or dephosphorylation at S137 during the first 60’ post LTP induction, we tested next whether this was also true for induction of NMDAR-dependent long-term depression (LTD, chemically induced). In contrast to NMDAR-dependent LTP, the loss of PFN2a did not prevent spine head plasticity (Figures 4B, C). A significant reduction in spine head diameter was also observed in neurons expressing any of the two phospho-mutants, however, spine shrinkage was reduced by 50% in case of the phospho-mimetic PFN2a S137D.

In summary, our results suggest a phosphorylation at the S137 side of PFN2a leads to a status-dependent regulation of dendritic spine actin dynamics in order to mediate synaptic plasticity mechanisms in dendritic spines of hippocampal neurons.

## 4 Discussion

The phosphorylation of PFN2a at multiple amino acid residues and the resulting modulation of PFN2a function in the cellular context by alteration of binding kinetics to other interaction partners has already been shown and described previously (Walter et al., 2020). However, whether the regulation of PFN2a function through phosphorylation plays an essential role for neuronal plasticity mechanisms could not yet be directly shown. Therefore, at first, to assess the general phosphorylation status of PFN2a phosphorylation, we used 2D gel electrophoresis to analyze hippocampal lysates. Expanding previous studies (Da Silva et al., 2003), also including work from our own lab (Schweinhuber et al., 2015), we could show that PFN2a is modified by three different degrees of phosphorylation *in vivo*. Although these results clearly confirm that PFN2a phosphorylation is of importance *in vivo*, they do not offer evidence on which amino acids of PFN2a are actually phosphorylated and how these phosphorylation states are characterized. Thus, we aimed at analyzing the role of PFN2a phosphorylation more directly, at individual known and previously described phosphorylation sites (Walter et al., 2020). We utilized phospho-mimetic (S137D) or phospho-deficient (S137A) PFN2a S137 mutants, and first analyzed their impact on basal dendritic spine properties and spine actin dynamics. Phosphorylation at S137 is closely localized to both, the PLP- as well as the PIP_2_-binding pockets of PFN2a and can thereby modulate binding characteristics to most PFN2a interaction partners, offering a high degree of regulatory potential (Walter et al., 2020). As described previously (Michaelsen et al., 2010a), we utilized an experimental knock-down/knock-in approach that depletes endogenous PFN2a and at the same time allows for the expression of RNAi resistant wildtype PFN2a (as a control), or the different S137 mutants. While the pure PFN2a knock-down had profound effects on both, basal actin dynamics as well as spine density and average spine head diameter, all these phenotypes could be completely rescued through re-introduction of wildtype PFN2a suggesting reconstitution with exogenous PFN2a was indeed possible. This allowed us to analyze the individual PFN2a S137 mutants in more detail. Interestingly, both mutants analyzed resulted in a significant decrease in the average diameter of the dendritic spine head upon substitution of endogenous PFN2a, similar to the phenotype in PFN2a knockdown neurons, indicating that phosphorylation at S137 is indeed important *in vivo* and that functional regulation of PFN2a *via* phosphorylation is required for the regulation of dendritic spine morphology.

In order to reveal potential mechanisms how PFN2a phosphorylation is involved in regulating spine morphology, we studied spine actin dynamics. Interestingly, with regard to a specific regulation *via* phosphorylation at S137, our results suggest that in hippocampal neurons, under basal conditions, S137 is predominantly phosphorylated since a substitution of endogenous PFN2a with phospho-deficient PFN2a S137A significantly altered spine actin dynamics while a substitution with phospho-mimetic PFN2a S137D had no influence on dynamic actin in spines. Hence, for basal actin dynamics, phosphorylation of PFN2a at S137 seems to be important.

Our results further indicate that this holds also true for activity-dependent modulation of spine actin dynamics in the early phase after LTP induction. 15′ following NMDAR-dependent cLTP induction, spine actin dynamics were similar to control neurons in cells expressing PFN2a S137D, whereas the activity-dependent modulation of dynamic actin in spines was impaired in neurons expressing PFN2a S137A. This suggests that phosphorylation of PFN2a at S137 is also needed for the modulation of dynamic actin during the early phase of synaptic plasticity. Interestingly, when structural plasticity was analyzed 60′ following NMDAR-dependent cLTP induction, spine head growth was prevented by the expression of phospho-deficient PFN2a S137A, but also phospho-mimetic PFN2a S137D. Thus, although basal actin dynamics as well as the early phase of synaptic plasticity were unaltered in neurons expressing the phospho-mimetic mutant, structural plasticity and a long-lasting, actin-dependent spine growth could not be induced. This suggests that sometime between 15′ and 60’ post LTP induction, S137 must be dephosphorylated for structural plasticity to occur.

Our experiments so far showed a crucial regulation of PFN2a function by phosphorylation under basal conditions as well as during LTP. We were therefore interested whether PFN2a might be also involved in NMDAR-dependent LTD. Interestingly, in PFN2a-deficient neurons, spine shrinkage 60’ following cLTD induction was similar to control cells. Spine shrinkage was also unaltered when substituting endogenous PFN2a with phospho-deficient PFN2a S137A. It was, however, reduced to ∼50% following substitution by phospho-mimetic PFN2a S137D. Hence, dephosphorylation of PFN2a seems to be important in a bidirectional manner in order to allow for spine structural changes to occur following LTP as well as LTD induction.

Overall, these results suggest that PFN2a phosphorylation is an essential mechanism to mediate PFN2a function in order to modulate spine actin dynamics in hippocampal neurons *in vivo* and propose that a regulation of PFN2a function through phosphorylation at S137 is crucial for an activity-dependent remodeling of spine actin dynamics during LTP and LTD. In general, our data lead to the following model: Under basal conditions, S137 seems to be phosphorylated. Hence, since previous studies showed that phosphorylation at S137 decreases PFN2a binding to both, PLP- as well as PIP_2_-binding partners (Walter et al., 2020), PFN2a might be kept in a state where the potential to boost actin dynamics (especially through interactions with PLP-containing ligands) is not fully exploited. In line with this, our results suggest that following induction of LTP, between 15′ and 60’ post-stimulus, S137 needs to be dephosphorylated which would, theoretically, provide more available PFN2a to PLP-ligands, thereby boosting F-actin polymerization. Further supporting this, the late phase of LTP is especially characterized by a shift in the F-to-G-actin equilibrium towards F-actin (Okamoto et al., 2004) which is believed to provide the core stability and strength needed for long-term dendritic spine growth through actin remodeling. In addition, previous work could show that an increase of F-actin polymerization alone can convert early-into late-LTP (Huang et al., 2013). Therefore, strengthening previous observations that a lack of PFN2a almost completely abolishes structural plasticity, PFN2a might be specifically recruited to boost F-actin dynamics during activity-dependent actin modifications following LTP induction through a targeted dephosphorylation of S137. Which kinases and phosphatases are mediating these regulations, however, remains speculative. So far, three kinases where shown to phosphorylate S137 at PFN2a: the protein kinase A (Schweinhuber et al., 2015), the ROCK II kinase (Witke et al., 1998) as well as the protein kinase C (which only phosphorylates PIP_2_-bound PFN2a—potentially as a mechanism to release it from the membrane) (Hansson et al., 1988; Singh et al., 1996). However, no phosphatase could be identified to reverse this modification up until now, at least not for PFN2a. For S137 of PFN1, it was shown that it is dephosphorylated by the protein phosphatase 1 (PP1) (Shao and Diamond, 2012). If PP1 is also able to dephosphorylate PFN2a S137 could not yet be confirmed. Our data, however, might indeed suggest such a model as, in line with our observations of phosphorylated S137 during early-LTP, PP1 is kept inactive during LTP (Blitzer et al., 1998) and *vice versa*, PP1 is activated following LTD induction (Mulkey et al., 1993; Thiels et al., 1998) which would fit to our hypothesis that during LTD, PFN2a S137 gets dephosphorylated. Intriguingly, a similar regulatory switch mechanism has already been described for PP1 and its modulation of Ca2+/calmodulin (CaM)-dependent protein kinase II activity during processes of functional plasticity, like LTP and LTD (Lisman and Zhabotinsky, 2001; Munton et al., 2004).

Taken together, our data show that targeted (de)phosphorylation of PFN2a at S137 modulates PFN2a function which is needed for bidirectional structural plasticity at dendritic spines following NMDAR-dependent LTP and LTD.

## Data Availability

The raw data supporting the conclusion of this article will be made available by the authors, without undue reservation.
