# Supplementary material for: Phosphorylation of the actin-binding protein profilin2a at S137 modulates bidirectional structural plasticity at dendritic spines
Source: Front Cell Dev Biol. 2023 Feb 15;11:1107380. doi: 10.3389/fcell.2023.1107380 (PMC9975505; doi:10.3389/fcell.2023.1107380)
Supplement: Supplementary file 1 [file Table1.docx]

# Supplementary Material

**Table S1 – Statistical Information.**

| **Figure** | **Treatment & Statistics Note that N = 3 for all experiments performed** | | | | |
| --- | --- | --- | --- | --- | --- |
| **Fig.1A** | **% of total PFN2a** | Spot 1 |  | 2.35 ± 2.13 % |  |
|  |  | Spot 2 |  | 32.76 ± 11.66 % |  |
|  | One-Way ANOVA | Spot 3 |  | 41.86 ± 9.22 % |  |
|  | Post-hoc Tuckey | p ≤ 0.05 vs Spot 1 |  |  |  |
|  |  | Spot 4 |  | 23.03 ± 1.80 % |  |
|  |  |  |  |  |  |
|  | **pPFN2a – PFN2a ratio** |  |  | 5.190 ± 1.50 |  |
|  |  |  |  |  |  |
| **Fig.1B** | **Turnover time** | Ctrl |  | 36.90 ± 2.30 s |  |
|  |  | PFN2a KD |  | 57.75 ± 3.65 s |  |
|  | One-Way ANOVA | p ≤ 0.001 vs Ctrl |  |  |  |
|  | Post-hoc Tuckey | PFN2a WT mod |  | 35.73 ± 3.01 s |  |
|  |  | p ≤ 0.001 vs PFN2a KD |  |  |  |
|  |  |  |  |  |  |
|  | **Dynamic fraction** | Ctrl |  | 0.82 ± 0.02 |  |
|  |  | PFN2a KD |  | 0.74 ± 0.03 |  |
|  | One-Way ANOVA | p ≤ 0.05 vs Ctrl |  |  |  |
|  | Post-hoc Tuckey | PFN2a WT mod |  | 0.80 ± 0.02 |  |
|  |  |  |  |  |  |
| **Fig.1C** | **Spine density** | Ctrl |  | 0.80 ± 0.04 per µm |  |
|  |  | PFN2a KD |  | 0.94 ± 0.04 per µm |  |
|  | One-Way ANOVA | p ≤ 0.05 vs Ctrl |  |  |  |
|  | Post-hoc Tuckey | PFN2a WT mod |  | 0.90 ± 0.03 per µm |  |
|  |  |  |  |  |  |
|  | **Spine head diameter** | Ctrl |  | 0.58 ± 0.02 µm |  |
|  |  | PFN2a KD |  | 0.49 ± 0.02 µm |  |
|  | One-Way ANOVA | p ≤ 0.01 vs Ctrl |  |  |  |
|  | Post-hoc Tuckey | PFN2a WT mod |  | 0.55 ± 0.01 µm |  |
|  |  | p ≤ 0.05 vs PFN2a KD |  |  |  |
|  |  |  |  |  |  |
| **Fig.1F** | **Spine head diameter** | Ctrl |  | 0.58 ± 0.02 µm |  |
|  |  | PFN2a KD |  | 0.49 ± 0.02 µm |  |
|  | One-Way ANOVA | p ≤ 0.05 vs PFN2a WT mod |  |  |  |
|  | Post-hoc Dunnett | PFN2a WT mod |  | 0.55 ± 0.01 µm |  |
|  |  | PFN2a S137A |  | 0.48 ± 0.02 µm |  |
|  |  | p ≤ 0.01 vs PFN2a WT mod |  |  |  |
|  |  | PFN2a S137D |  | 0.48 ± 0.01 µm |  |
|  |  | p ≤ 0.01 vs PFN2a WT mod |  |  |  |
|  |  |  |  |  |  |
| **Fig.1G** | **Mean fluor. intensity** | PFN2a WT mod |  | 1.00 ± 0.09 |  |
|  |  | PFN2s S137A |  | 0.99 ± 0.16 |  |
|  |  | PFN2a S137D |  | 1.03 ± 0.12 |  |
|  |  |  |  |  |  |
| **Fig.2A** | Two-Way ANOVA | Mutant vs Mutant | F = 20.7 df = 1 p < 0.0001 | | |
|  |  |  |  |  |  |
| **Fig.2B** | Two-Way ANOVA | Mutant vs Mutant | F = 8.65 df = 1 p = 0.0041 | | |
|  |  |  |  | | |
| **Fig.2C** | Two-Way ANOVA | Mutant vs Mutant | F = 0.0097 df = 1 p = 0.9217 | | |
|  |  |  |  | | |
| **Fig.2D** | **Turnover time** | PFN2a WT mod |  | 35.73 ± 3.01 s |  |
|  |  | PFN2a KD |  | 57.75 ± 3.65 s |  |
|  | One-Way ANOVA | p ≤ 0.001 vs PFN2a WT mod |  |  |  |
|  | Post-hoc Dunnett | PFN2a S137A |  | 26.53 ± 2.78 s |  |
|  |  | PFN2a S137D |  | 34.29 ± 2.49 s |  |
|  |  |  |  |  |  |
| **Fig.2E** | **Dynamic fraction** | PFN2a WT mod |  | 0.80 ± 0.02 |  |
|  |  | PFN2a KD |  | 0.74 ± 0.03 |  |
|  | One-Way ANOVA | PFN2a S137A |  | 0.84 ± 0.02 |  |
|  | Post-hoc Dunnett | PFN2a S137D |  | 0.80 ± 0.03 |  |
|  |  |  |  |  |  |
| **Fig.3A** | Two-Way ANOVA | Unstimulated vs cLTP | F = 7.51 df = 1 p = 0.0077 | | |
|  |  |  |  |  |  |
| **Fig.3B** | Two-Way ANOVA | Unstimulated vs cLTP | F = 0.382 df = 1 p = 0.5386 | | |
|  |  |  |  |  |  |
| **Fig.3C** | Two-Way ANOVA | Unstimulated vs cLTP | F = 1.79 df = 1 p = 0.1851 | | |
|  |  |  |  |  |  |
| **Fig.3D** | Two-Way ANOVA | Unstimulated vs cLTP | F = 6.18 df = 1 p = 0.0152 | | |
|  |  |  |  |  |  |
| **Fig.3E** | **Turnover time** | PFN2a WT mod |  | 35.73 ± 3.01 s |  |
|  |  | PFN2a KD |  | 57.75 ± 3.65 s |  |
|  | T-TEST | PFN2a S137A |  | 26.53 ± 2.78 s |  |
|  |  | PFN2a S137D |  | 34.29 ± 2.49 s |  |
|  |  |  |  |  |  |
|  |  | PFN2a WT mod LTP |  | 29.65 ± 3.35 s |  |
|  |  | PFN2a KD LTP |  | 48.83 ± 4.34 s |  |
|  |  | PFN2a S137A LTP |  | 35.43 ± 3.63 s |  |
|  |  | PFN2a S137D LTP |  | 30.97 ± 3.37 s |  |
|  |  |  |  |  |  |
| **Fig.3F** | **Dynamic fraction** | PFN2a WT mod |  | 0.80 ± 0.02 |  |
|  |  | PFN2a KD |  | 0.74 ± 0.03 |  |
|  | T-TEST | PFN2a S137A |  | 0.84 ± 0.02 |  |
|  |  | PFN2a S137D |  | 0.80 ± 0.03 |  |
|  |  |  |  |  |  |
|  |  | PFN2a WT mod LTP |  | 0.88 ± 0.03 |  |
|  |  | p = 0.004 vs PFN2a WT mod |  |  |  |
|  |  | PFN2a KD LTP |  | 0.69 ± 0.02 |  |
|  |  | PFN2a S137A LTP |  | 0.86 ± 0.02 |  |
|  |  | PFN2a S137D LTP |  | 0.90 ± 0.03 |  |
|  |  | p = 0.024 vs PFN2a S137D |  |  |  |
|  |  |  |  |  |  |
| **Fig.4A** | **Spine head diameter** | PFN2a KD |  | 0.49 ± 0.02 µm |  |
|  |  | PFN2a WT mod |  | 0.55 ± 0.01 µm |  |
|  | T-TEST | PFN2a S137A |  | 0.48 ± 0.02 µm |  |
|  |  | PFN2a S137D |  | 0.48 ± 0.01 µm |  |
|  |  |  |  |  |  |
|  |  | PFN2a KD LTP |  | 0.42 ± 0.01 µm |  |
|  |  | p = 0.0076 vs PFN2a KD |  |  |  |
|  |  | PFN2a WT mod LTP |  | 0.69 ± 0.02 µm |  |
|  |  | p ≤ 0.0001 vs PFN2a WT mod |  |  |  |
|  |  | PFN2a S137A LTP |  | 0.51 ± 0.01 µm |  |
|  |  | PFN2a S137D LTP |  | 0.51 ± 0.01 µm |  |
|  |  |  |  |  |  |
| **Fig.4B** | **Spine head diameter** | PFN2a KD |  | 0.49 ± 0.02 µm |  |
|  |  | PFN2a WT mod |  | 0.55 ± 0.01 µm |  |
|  | T-TEST | PFN2a S137A |  | 0.48 ± 0.02 µm |  |
|  |  | PFN2a S137D |  | 0.48 ± 0.01 µm |  |
|  |  |  |  |  |  |
|  |  | PFN2a KD LTD |  | 0.39 ± 0.01 µm |  |
|  |  | p ≤ 0.0001 vs PFN2a KD |  |  |  |
|  |  | PFN2a WT mod LTD |  | 0.45 ± 0.02 µm |  |
|  |  | p ≤ 0.0001 vs PFN2a WT mod |  |  |  |
|  |  | PFN2a S137A LTD |  | 0.39 ± 0.01 µm |  |
|  |  | p = 0.001 vs PFN2a S137A |  |  |  |
|  |  | PFN2a S137D LTD |  | 0.43 ± 0.01 µm |  |
|  |  | p = 0.0174 vs PFN2a S137D |  |  |  |
|  |  |  |  |  |  |
|  | **Spine shrinkage** | PFN2a KD |  | 20.98 ± 1.51 % |  |
|  |  | p ≤ 0.01 vs PFN2a S137D |  |  |  |
|  | One-Way ANOVA | PFN2a WT mod |  | 19.16 ± 2.79 % |  |
|  | Post-hoc Dunnett | p ≤ 0.05 vs PFN2a S137D |  |  |  |
|  |  | PFN2a S137A |  | 19.80 ± 2.09 % |  |
|  |  | p ≤ 0.01 vs PFN2a S137D |  |  |  |
|  |  | PFN2a S137D |  | 10.25 ± 1.67 % |  |
